# CCR7 Sulfotyrosine Enhances CCL21 Binding

**DOI:** 10.3390/ijms18091857

**Published:** 2017-08-25

**Authors:** Andrew J. Phillips, Deni Taleski, Chad A. Koplinski, Anthony E. Getschman, Natasha A. Moussouras, Amanda M. Richard, Francis C. Peterson, Michael B. Dwinell, Brian F. Volkman, Richard J. Payne, Christopher T. Veldkamp

**Affiliations:** 1 Department of Chemistry, University of Wisconsin-Whitewater, Whitewater, WI 53190, USA; andrew.phillips@unmc.edu (A.J.P.); RichardAM08@uww.edu (A.M.R.); 2 School of Chemistry, University of Sydney, Sydney 2006, Australia; deni.taleski@gmail.com (D.T.); richard.payne@sydney.edu.au (R.J.P.); 3 Department of Biochemistry, Medical College of Wisconsin, Milwaukee, WI 53226, USA; ckoplinski@mcw.edu (C.A.K.); agetschman@mcw.edu (A.E.G.); fpeterso@mcw.edu (F.C.P.); bvolkman@mcw.edu (B.F.V.); 4 Department of Microbiology and Immunology, Medical College of Wisconsin, Milwaukee, WI 53226, USA; nmoussouras@mcw.edu (N.A.M.); mdwinell@mcw.edu (M.B.D.)

**Keywords:** chemokines, chemokine receptors, NMR, sulfotyrosine, CCL21, CCL19, CCR7, cancer metastasis, posttranslational modification

## Abstract

Chemokines are secreted proteins that direct the migration of immune cells and are involved in numerous disease states. For example, CCL21 (CC chemokine ligand 21) and CCL19 (CC chemokine ligand 19) recruit antigen-presenting dendritic cells and naïve T-cells to the lymph nodes and are thought to play a role in lymph node metastasis of CCR7 (CC chemokine receptor 7)-expressing cancer cells. For many chemokine receptors, N-terminal posttranslational modifications, particularly the sulfation of tyrosine residues, increases the affinity for chemokine ligands and may contribute to receptor ligand bias. Chemokine sulfotyrosine (sY) binding sites are also potential targets for drug development. In light of the structural similarity between sulfotyrosine and phosphotyrosine (pY), the interactions of CCL21 with peptide fragments of CCR7 containing tyrosine, pY, or sY were compared using protein NMR (nuclear magnetic resonance) spectroscopy in this study. Various N-terminal CCR7 peptides maintain binding site specificity with Y8-, pY8-, or sY8-containing peptides binding near the α-helix, while Y17-, pY17-, and sY17-containing peptides bind near the N-loop and β3-stand of CCL21. All modified CCR7 peptides showed enhanced binding affinity to CCL21, with sY having the largest effect.

## 1. Introduction

Chemokines are small, secreted proteins that traffic immune cells in the body through functioning as chemoattractants [1]. There are approximately 50 chemokines and 20 chemokine receptors, of which many are involved in diseases, including inflammatory diseases like rheumatoid arthritis, viral diseases like human immunodeficiency virus-1 (HIV-1)/acquired immune deficiency syndrome (AIDS), and cancer metastasis [2]. The chemokine receptor CCR7 and its chemokine ligands CCL21 and, less so, CCL19, recruit circulating metastatic cancer cells to lymphatic tissue [3,4,5,6]. Chemokine receptors, like CCR7, are integral membrane proteins belonging to the rhodopsin-like or class A family of G protein-coupled receptors [1]. After synthesis, many chemokine receptors are post-translationally modified in the Golgi apparatus by tyrosylprotein sulfotransferase (TPST) enzymes, including the receptors CCR2, CCR3, CCR5, CCR8, CXC chemokine receptor 3 (CXCR3), CXCR4, and CX_3_C chemokine receptor 1 (CX_3_CR1) [7,8,9,10,11,12,13]. Tyrosine sulfation of chemokine receptor N-termini generally enhances affinity and encodes specificity between the chemokine and the receptor [10,13,14,15,16,17,18,19]. Chemokine receptor activation by balanced chemokine ligands generally leads to intracellular signaling through G protein- and β-arrestin-dependent pathways. Importantly, receptor activation by more than one or naturally modified ligands can lead to the reduction or absence of either pathway with a striking biological effect, which is described as “ligand” bias [20]. Lefkowitz et al. first showed CCR7 to a have biased signaling response to its two native ligands CCL21 and CCL19 [21].

Depending on the chemokine and the receptor, the receptor activation often results in intracellular signaling pathways involving the activation of intracellular kinases, of which many are serine/threonine kinases and some are tyrosine kinases [20]. Since TPST enzymes are located in the Golgi and modify tyrosine residues in certain integral membrane and secreted proteins [7], while tyrosine kinases are cytosolic enzymes, sulfotyrosine (sY) and phosphotyrosine (pY) are spatially segregated to different cellular locations. Given the single atom difference between sY and pY, it seems appropriate to ask if there are significant functional differences between the two posttranslational modifications. Others have investigated this question and have observed differing results. Hirudin, an anticoagulant, normally contains a sY at position 63 that is important for thrombin binding [22]. Replacement of sY63 in hirudin with pY showed no difference in thrombin binding and inhibition of coagulation, suggesting that sY and pY are potentially interchangeable posttranslational modifications [22]. However, sY and pY modifications are not equivalent in other systems. Sulfation of tyrosines in the N-termini of CCR5, a co-receptor for HIV-1, facilitates HIV-1 cell entry [23,24] and chemokine binding [25]. Peptides fragments of the CCR5 N-terminus containing sY residues bound envelope glycoprotein (gp)-120/CD4 complexes, while those containing tyrosine or pY residues did not [26]. Other examples have shown that sY and pY modifications are somewhat interchangeable, with SH2 domains binding much less tightly to sY versus native pY containing ligands [27,28]. Recent molecular dynamics studies investigating the structure of the chemokine CXC chemokine ligand 12 (CXCL12) bound to the sY-modified CXCR4 N-terminus predicted that substituting pY would increase the affinity of the CXCL12 and CXCR4 interaction [29]. Stone and colleagues have also demonstrated that inorganic phosphate can modulate the recognition of sulfotyrosine residues in a CCR2 receptor mimic by the CCL2 chemokine [30]. These reports suggest there are no particular trends and comparisons of the effects of sY and pY posttranslational modifications on binding that would need to be carried out on a case by case basis.

Chemokines are hypothesized to bind to and activate their receptors through a two site-two state binding and activation model [31,32]. The extracellular, sulfotyrosine-containing N-terminus of the chemokine receptor binds to the chemokine domain first, site one, which is followed by binding of the chemokine N-terminus to a second site on the receptor that results in a receptor conformational change and in activation. Due to their demonstrated role in receptor binding and specificity, we and others have focused on using the sY-chemokine interaction as a way to identify and target druggable “hotspots” on the chemokine. The CXCR4 sY21 and sY12 binding sites on CXCL12 have successfully been targeted with inhibitory small molecules [33,34,35,36,37,38]. These inhibitors are presumed to disrupt CXCL12’s interaction with the CXCR4 N-terminus, and this has prompted a comparison of sulfotyrosine and phosphotyrosine modifications in the context of CCL21 binding to its receptor, CCR7. The CCR7 N-terminus contains two tyrosines at positions 8 and 17 which are predicted to be sulfated by the bioinformatics program SulfoSite [39], while only tyrosine 8 is predicted to be sulfated by Sulfinator [40]. Here we use sY-containing CCR7 N-terminal peptides to identify putative sY8 and sY17 binding regions on CCL21 and compare the impact of tyrosine sulfation versus phosphorylation or the absence of posttranslational modification on binding specificity and affinity.

## 2. Results

### 2.1. A CCR7 N-Terminal Peptide Binds CCL21

**Figure 1:**
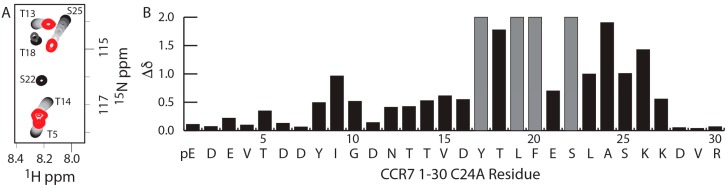
CCL21-induced chemical shift perturbations in the CCR7 N-terminus including tyrosine 8 and 17 and adjacent amino acids. (**A**) ^15^N-^1^H HSQC spectra of 200 μM U-^15^N/^13^C CCR7 1–30 C24A in the absence (black) and with increasing concentrations of CCL21 (grays) at 1:2.5 CCR7:CCL21 molar ratio (red); (**B**) CCL21-induced CCR7 1–30 C24A chemical shift perturbations. A portion of glutamine 1 of CCR7 1–30 C24A spontaneously reacts to form pyroglutamate; hence, the pE or pyroglutamate label at position 1. CCR7 residues that broadened beyond detection during the titration are indicated in gray with a chemical shift of 2.

**Figure 2:**
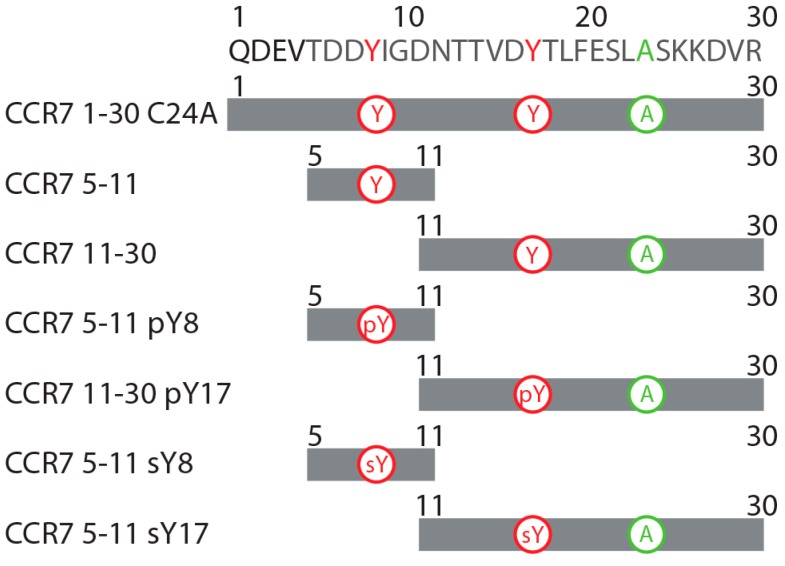
Schematic of the various CCR7 N-terminal peptides used in this study. CCR7 peptides correspond to the sequence of the mature CCR7 N-terminus. Those peptides including residue 24 have a C24A mutation to prevent oxidative peptide dimer formation. Tyrosine posttranslational modifications are as indicated.

As chemokines are thought to bind and activate their receptors through a two-step, two-site binding and activation model [31,32], peptides corresponding to the receptor N-terminus have historically been used to mimic the site 1 interaction. To determine which residues in the CCR7 N-terminus may be important for binding to CCL21 as a part of site 1, a titration of a uniformly ^15^N-labeled CCR7 1–30 C24A peptide with increasing concentrations of CCL21 was monitored using ^15^N-^1^H heteronuclear single quantum coherence (HSQC) spectroscopy (Figure 1A). The CCR7 peptide contained a C24A mutation to prevent oxidative dimer formation. CCL21 caused chemical shift perturbations in the CCR7 N-terminus that included residues adjacent to both Y8 and Y17 (Figure 1B). Given that sulfotyrosines in other chemokine receptors increase affinity for the chemokine ligand [10,13,14,15,16,17,18,19], these CCR7 chemical shift perturbations guided our design and synthesis of CCR7 N-terminal peptides to investigate tyrosine modifications at Y8 or Y17 (Figure 2). The Y8 modification was explored using CCR7 peptides containing residues 5–11 and either Y8, pY8, or sY8, while the Y17 modification was studied using CCR7 peptides containing residues 11–30 and either Y17, pY17, or sY17.

### 2.2. CCR7 N-Terminal Peptides Maintain Binding Site Specificity upon Tyrosine Modification

**Figure 3:**
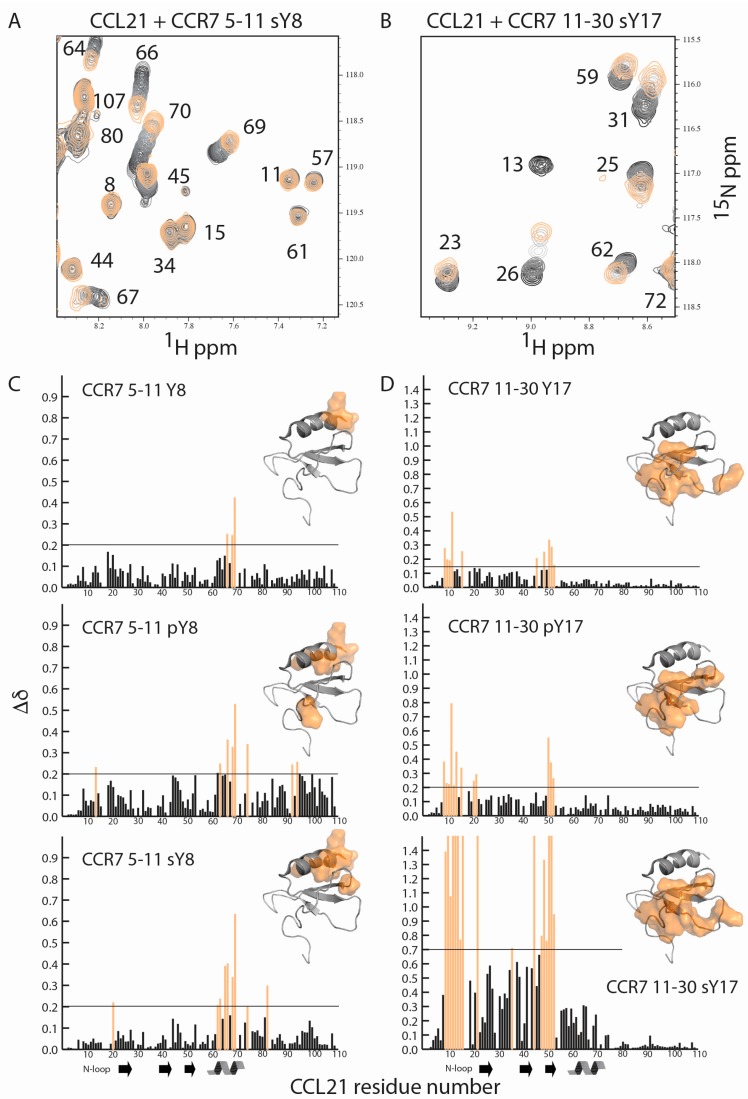
CCR7 N-terminal peptides maintain binding site specificity regardless of tyrosine modification. (**A**) ^15^N-^1^H HSQC spectra of 100 μM U-^15^N CCL21 (black) with increasing concentrations of CCR7 5–11 sY8 in gray and orange (1:10 molar ratio); (**B**) ^15^N-^1^H HSQC spectra of 100 µM U-^15^N CCL21 (black) with increasing concentrations of CCR7 11–30 sY17 in gray and orange (1:10 molar ratio); (**C**) CCL21 chemical shift perturbations and chemical shift mapping for CCR7 5–11 Y8, pY8, and sY8; (**D**) CCL21 chemical shift perturbations and chemical shift mapping for CCR7 11–30 Y17, pY17, and sY17. Residues with a chemical shift perturbation above 1.4 broadened beyond detection during the titration indicative of intermediate exchange. For clarity residues with significant chemical shift perturbations that were mapped onto the structure of CCL21-lacking residues 71–111, an extended and unstructured C-terminus, which was present [41]. CCL21 prolines and any unobservable backbone amides have chemical shift perturbations of zero.

Site one interactions form long, extended binding epitopes that can encompass significant amounts of the chemokine surface. To observe the important differences between modifications, U-^15^N labeled CCL21 was titrated with the various CCR7 N-terminal peptides and NMR experiments performed. Regardless of the tyrosine modification on the CCR7(5–11) or CCR7(11–30) N-terminal peptides, binding site specificity on CCL21 was maintained, with CCR7 5–11 peptides binding alongside the alpha helix and CCR7 11–30 peptides binding to the N-loop and β3-strand of CCL21. Representative data showing overlays from a portion of the ^15^N-^1^H HSQC spectra of CCL21 titrated with increasing concentrations of CCR7 5–11 sY8 or CCR7 11–30 sY17 are shown in Figure 3A,B. With the exception of the CCR7 11–30 sY17 peptide, binding was in fast exchange and CCL21 assignments could be transferred by inspection. The majority of CCL21 residues bound in fast exchange upon titration with the CCR7 11–30 sY17 peptide, but a small number of residues broadened beyond detection during the titration, indicative of intermediate exchange. Residues that broadened beyond detection are indicated with a maximum chemical shift perturbation value in Figure 3D. A plot of CCL21 chemical shift perturbations at the highest CCL21: peptide molar ratio of 1:10 for peptides containing sY, or 1:30 for peptides containing Y or pY, is shown in Figure 3C for the CCR7 5–11 peptides and in Figure 3D for the CCR7 11–30 peptides. Identical residues and/or similar regions of the CCL21 structure show perturbations suggesting binding site specificity is retained regardless of the type of or the absence of tyrosine modification.

### 2.3. Sulfotyrosine or Phosphotyrosine Modification Increase the Affinity of CCR7 N-Terminal Peptides for CCL21

**Figure 4:**
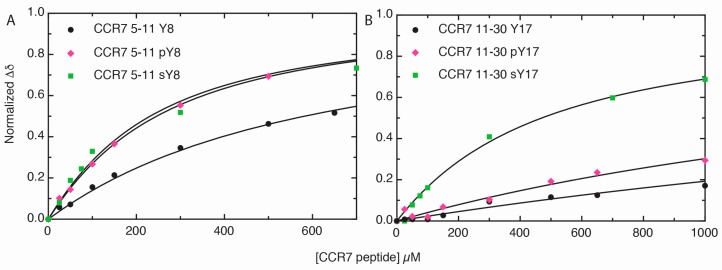
Tyrosine modification increases affinity of CCR7 N-terminal peptides for CCL21. Nonlinear fitting of residues with significant chemical shift perturbations was used for dissociation constant (K_d_) determination. Nonlinear fitting also provided the maximum chemical shift, which was normalized to 1 in the figure for better visual comparison. (**A**) Nonlinear fitting of normalized, combined amide chemical shift perturbations for CCL21 K69, a representative residue, plotted versus CCR7 peptide concentration. For this residue only, the K_d_ values were as follows: CCR7 5–11 K_d_ = 530 ± 40 µM, CCR7 5–11 pY8 K_d_ = 240 ± 90 µM, CCR7 5–11 sY8 K_d_ = 180 ± 40 µM; (**B**) Nonlinear fitting of normalized, combined amide chemical shift perturbations for CCL21 A53, a representative residue, plotted versus CCR7 peptide concentration. For this residue only, the K_d_ values were as follows: CCR7 11–30 K_d_ = 4100 ± 900 µM, CCR7 11–30 pY17 K_d_ = 2200 ± 200 µM, and CCR7 11–30 sY17 K_d_ = 420 ± 80 µM.

As the size of a chemical shift perturbation does not necessarily correlate with affinity, concentration-dependent CCL21 chemical shift perturbations were used to calculate the binding affinity of CCL21 for the various CCR7 peptides. Figure 4 shows representative nonlinear fitting data and dissociation constant (K_d_) values for the representative residue only. Figure 4A shows the nonlinear fitting of normalized, combined amide-proton chemical shift perturbations of a representative CCL21 residue, K69, plotted versus CCR7 peptide concentrations for CCR7 5–11, CCR7 5–11 pY8, or CCR7 5–11 sY8. The nonlinear fitting of normalized, combined amide-proton chemical shift perturbations for CCL21 A53, a representative residue, plotted versus CCR7 peptide concentration for CCR7 11–30, CCR7 11–30 pY17, or CCR7 sY17 is shown in Figure 4B. Average dissociation constants obtained from the non-linear fitting of chemical shift changes for all CCL21 residues with significant peptide perturbations are reported in Table 1. Sulfated CCR7 N-terminal peptides had the highest affinity for CCL21, followed by those containing pY and, finally, the peptides with unmodified tyrosine residues. For the CCR7 5–11 peptides, a pY modification increased affinity to nearly the same extent as sY, 240 ± 60 µM versus 140 ± 40 µM K_d_ values, respectively. However, the pY17 modification in the CCR7 11–30 peptide (K_d_ = 1700 ± 400 µM) did not increase affinity to CCL21 as dramatically as a sY17 modification (K_d_ = 480 ± 70 µM). Interestingly, the CCR7 5–11 peptides exhibited higher affinities for CCL21 than their corresponding CCR7 11–30 counterparts, despite inducing smaller chemical shift perturbations.

## 3. Discussion

**Figure 5:**
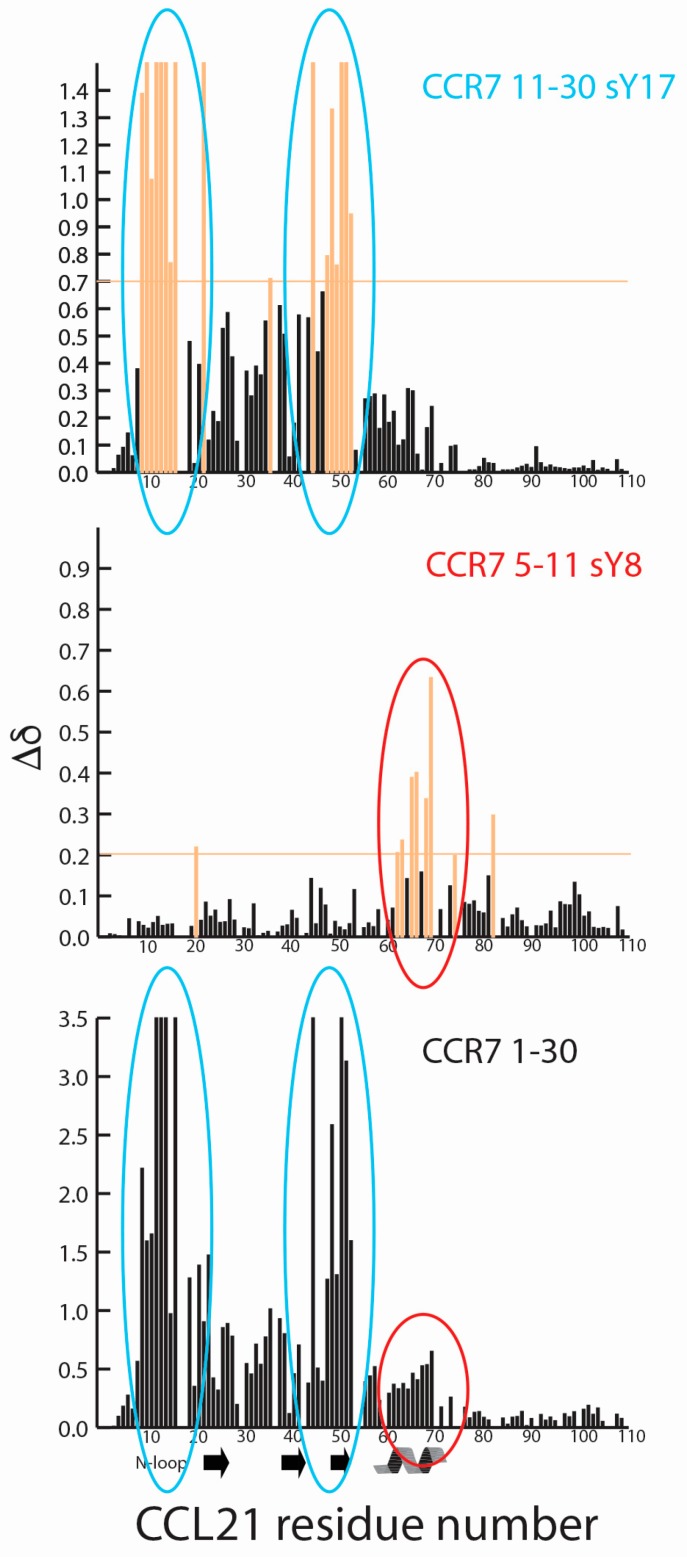
CCR7 site 1 is potentially modular with respect to CCL21 binding. CCL21 chemical shift perturbations are shown for CCR7 11–30 sY17 (top), CCR7 5–11 sY8 (middle), and CCR7 1–30 (bottom), adapted from Love et al. [41]. For CCR7 11–30 sY17 and CCR7 1–30, residues whose signal broadens beyond detection during titration are indicated with the highest chemical shift perturbation value. CCR7 11–30 sY17 and other CCR7 11–30 peptides induce chemical shift perturbations in the N-loop and β3 strand (circled in blue) with similar perturbations observed for CCR7 1–30, as indicated by blue ovals. CCR7 5–11 sY8 and other CCR7 5–11 peptides induce chemical shift perturbations in CCL21’s α-helix with potentially similar perturbations observed for CCR7 1–30, as indicated with the red ovals.

Our previous NMR studies defined the 3D structure of full-length CCL21 and mapped the interaction surface of a peptide corresponding to the N-terminal 30 residues of CCR7 [41]. The unsulfated CCR7 1–30 peptide induced significant chemical shift perturbations in the N-loop and β3 strand of CCL21. We separated the CCR7 N-terminal domain into two fragments corresponding to residues 5–11 and 11–30, each of which contained a tyrosine that may be a substrate for sulfation by TPST enzymes. While each peptide interacted with CCL21, the shifts induced by CCR7 11–30 binding were very similar to the pattern observed in the previous titration with CCR7 1–30. The CCR7 5–11 peptides induced smaller chemical shifts in the α-helix of CCL21. Small chemical shifts in the α-helix of CCL21 were also observed with unsulfated CCR7 1–30, but these shifts were dwarfed by those in the N-loop and the β3 strand of CCL21. A comparison of CCL21 chemical shift perturbations for CCR7 5–11 sY8, CCR7 11–30 sY17, and CCR7 1–30 in Figure 5 indicates that CCR7 5–11 and CCR7 11–30 perturbations could potentially be found in those observed for the longer CCR7 1–30 peptide. This suggests that the CCR7 site 1 interaction with CCL21 may be somewhat modular as it can be dissected into two parts, potentially suggesting that small molecule ligands could be developed to target each part and later linked. Also, as the CCR7 5–11 peptides all have higher affinity for CCL21 than the correspondingly modified CCR7 11–30 peptides (Table 1), CCR7 residues 5–11 may contribute significantly to CCL21 recognition. Phosphorylation or sulfation of either tyrosine 8 or 17 enhanced the binding of CCR7 N-terminal receptor peptides to CCL21, but sulfation of tyrosine 17 had the largest effect, increasing the affinity of CCR7 11–30 by more than 10-fold (Table 1). While there is no consensus sequence for tyrosine sulfation, proximity to one or more acidic amino acids increases the likelihood of sulfation [39,40]. Tyrosine 17 is next to aspartate 16 suggesting it may be a poorer TPST substrate than tyrosine 8, which follows aspartate 6 and 7. This is interesting as sulfotyrosine 17 has the greater impact in this analysis.

Binding of CCL19 or CCL21 to CCR7 leads to differential activation of GRKs and recruitment of β-arrestin, resulting in different cellular responses for the two chemokine ligands [21,42]. For example, only CCL19 activation of CCR7 results in receptor internalization and desensitization [21,42]. A truncated version of CCL21, in which CCL21’s unique, glycosaminoglycan-binding C-terminal tail has been proteolytically removed by plasmin, also results in signaling that is unique compared to CCL19 and full length CCL21 [43,44,45,46]. This ligand bias likely results from different CCR7 conformations in the presence of CCL19 and either full length or truncated CCL21 [43], but tyrosine sulfation in CCR7 could also be a contributing factor. While we have only investigated the interaction of CCL21 with CCR7 sulfopeptides here, Stone and colleagues have shown for CCR3 N-terminal peptides that different receptor tyrosine sulfation patterns impact which of the three chemokine ligands, CCL11, CCL24, or CCL26, the receptor prefers to bind [13,19]. It is plausible that that the degree of tyrosine sulfation or the ratio of sY8 to sY17 may impact affinity of CCR7 for its various ligands and thereby contribute to ligand bias. It should be noted that we attempted to use sodium chlorate, which has been reported to inhibit TPSTs and thus tyrosine sulfation [7], to determine if reducing CCR7 tyrosine sulfation impacted CCR7 signaling. However, treatment with sodium chlorate altered CCR7 surface expression levels, making results uninterpretable.

In addition to potential tyrosine sulfation, CCR7 is also posttranslationally modified through glycosylation. Sixt and colleagues report that polysialic acid is essential for the activation of CCR7 in dendritic cells by full length CCL21, while CCL19 and C-terminally truncated CCL21 can activate CCR7 independent of the polysialylation status of the receptor [47]. Legler and colleagues have reported distinct *N*-glycosylation patterns capped with sialic acid for specific cell types, for example B-cells and expanded T-cells, and that glycosylation inhibits chemokine activation of CCR7 unless removed by glycosidases [46]. One of the *N*-glycosylation and polysialylation sites is N12 of the mature CCR7 N-terminus (N36 if the signal sequence is included in the residue numbering) [46,47]. Interestingly, the CCR7 5–11 sY8 peptide induced chemical shifts located in the α-helix of CCL21 that are similar to those induced by polysialic acid as seen by Sixt and colleagues [47]. This further suggests a possible role for receptor sulfotyrosine in CCR7 ligand bias.

Here we show that that sulfotyrosine or phosphotyrosine increase the affinity of N-terminal CCR7 peptides for CCL21 with the sulfotyrosine modification having the largest effect. We also observe that CCR7 peptide binding specificity remains the same regardless of whether tyrosine residues are modified or not. Given that ligand bias is observed in CCR7 signaling and given the impact of posttranslational modifications like glycosylation or polysialylation [46,47], we hypothesize that the sulfotyrosine modification will also have an impact on biased signaling. A better understanding of biased signaling in CCR7 could allow for the selective targeting of only CCL19 or CCL21 signaling, which may be therapeutically beneficial for various disease states. Future studies will continue to focus on the impact of CCR7 N-terminal posttranslational modifications on binding to CCL21 and will also incorporate C-terminally truncated CCL21. These future studies will also seek to assess whether TPSTs can sulfate CCR7 Y8 and Y17 and move toward the inclusion of the full length CCR7 receptor through incorporating cell-based assays.

## 4. Materials and Methods

### 4.1 Recombinant Protein Purification and Peptide Synthesis

CCL21 was expressed and purified as previously described [48]. CCR7 1–30 C24A (QDEVTDDYIGDNTTVDYTLFESLASKKDVR), a peptide corresponding to the sequence of the N-terminus of mature CCR7 [49] (with the exception of a C24A mutation to prevent oxidative dimer formation), was expressed recombinantly and purified as follows. DNA coding for a SMT3-CCR7 1–30 C24A fusion was cloned into the BamHI and HindIII sites of pQE30. This pQE30-SMT3-CCR7 1–30 C24A was transformed into SG13009 [pREP4] *E. coli*. Cells were grown at 37 °C in 1L of U-^15^N/^13^C M9 minimal media to an optical density at 600 nm (OD_600_) of 0.6, at which time expression was induced with 1 mM isopropyl β-d-1-thiogalactopyranoside. After 5 h, cell pellets were collected by centrifugation (3000× *g* for 30 min) and stored at −80 °C until processing. Cells were resuspended and lysed by sonication in buffer A (50 mM sodium phosphate, 300 mM sodium chloride, 10 mM imidazole, pH 8.0) containing 0.1% (*v/v*) β-mercaptoethanol and 1 mM phenylmethanylsulfonyl fluoride. The lysate was clarified by centrifugation (15,000× *g* for 15 min) and the supernatant containing the His_6_-SMT3-CCR7 1–30 C24A fusion was applied to 2 mL of His60 nickel resin. The column was washed with 40 mL of buffer A and eluted with buffer B (50 mM sodium phosphate, 300 mM sodium chloride, 500 mM imidazole, pH 8.0). The eluent containing the His_6_-SMT3-CCR7 1–30 C24A fusion was dialyzed (MWCO 3,000) twice against 4 L of 20 mM TRIS, pH 8.0, at 4 °C with stirring. The dialysate was transferred to a 50 mL conical vial and digested with 400 μg of His_6_-Ubiquitin like protease 1 (His_6_-ULP1) with stirring at 4 °C until complete cleavage of the His_6_-SMT3-CCR7 1–30 C24A fusion was achieved based on SDS-PAGE. To separate the His_6_-ULP1 and His_6_-SMT3 from CCR7 1–30 C24A, the digestion was applied to 2 mL of His60 nickel resin. The column flow through and one 10 mL buffer A contained the CCR7 1–30 C24A was filtered (0.2 μm), and CCR7 1–30 C24A was further purified by reverse-phase HPLC using a 30 min 0–70% (*v/v*) acetonitrile gradient in aqueous 0.1% (*v/v*) triflouroacetic acid.

CCR7 sulfotyrosine-containing peptides (CCR7 5–11 sY8, NH_2_-TDDsYIGD-CONH_2_ and CCR7 11–30 sY17, NH_2_-DNTTVDsYTLFESLASKKDVR-CONH_2_) were synthesized and purified as previously described [12]. CCR7 phosphotyrosine-containing peptides (CCR7 5–11 pY8, NH_2_-TDDpYIGD-CONH_2_ and CCR7 11–30 pY17, NH_2_-DNTTVDpYTLFESLASKKDVR-CONH_2_) and CCR7 peptides lacking a posttranslational modification (CCR7 5–11 Y8, NH_2_-TDDYIGD-CONH_2_ and CCR7 11–30 Y17, NH_2_-DNTTVDYTLFESLASKKDVR-CONH_2_) were purchased from a commercial vendor.

### 4.2 Protein NMR

NMR spectroscopic data were collected at the NMR facility at the Medical College of Wisconsin on a Bruker Avance 600 MHz spectrometer equipped with ^1^H/^13^C/^15^N Cryoprobe^®^ at 25 °C. Chemical shift assignments for CCR7 1–30 C24A (H, N, C, C^α^, and most side chain carbons) were determined by standard techniques [50] using 1.05 mM U-^15^N/^13^C labeled CCR7 1–30 C24A in NMR buffer (25 mM deuterated MES, 10% D_2_O, 0.2% NaN_3_, pH 6.0). To determine CCR7 N-terminal residues likely involved in CCL21 binding, 200 μM U-^15^N/^13^C CCR7 1–30 C24A in NMR buffer was monitored using ^15^N-^1^H HSQC spectra while titrating with increasing CCL21 concentrations (molar ratios of 1:0, 1:0.25, 1:0.5, 1:0.75, 1:1, 1:1.5, 1:2 and 1:2.5 of CCR7:CCL21). Under these conditions the majority of CCR7 residues bind CCL21 in fast exchange, allowing for chemical shift assignments to be transferred by inspection, while some residues broadened beyond detection during the titration.

To identify the impact of CCR7 tyrosine modification on CCL21 binding, 100 μM of U-^15^N CCL21 in NMR buffer was titrated with increasing concentrations of the indicated CCR7 peptides and monitored using ^15^N-^1^H HSQC spectra, as previously described [51,52]. Molar ratio for CCL21 to CCR7 5–11, CCR7 5–11 pY8, CCR7 11–30, or CCR7 11–30 pY17 were as follows: 1:0, 1:0.25, 1:0.5, 1:1, 1:1.5, 1:3, 1:6.5, 1:10, 1:15, 1:25, and 1:30. Molar ratios for CCL21 to CCR7 5–11 sY8 or CCR7 11–30 sY17 were as follows: 1:0, 1:0.25, 1:0.5, 1:0.75, 1:1, 1:3, 1:7, and 1:10. Under these conditions most CCL21 residues bind the various CCR7 peptides in fast exchange, allowing chemical shift assignments [41] to be transferred by inspection. Combined amide chemical shift perturbations (Δδ) were computed as [(5Δδ_H_)^2^+(Δδ_N_)^2^]^1/2^, where Δδ_H_ and Δδ_N_ are the changes in backbone amide ^1^H and ^15^N chemical shifts in ppm, respectively. Dose-dependent changes in chemical shift perturbations upon titration with the various CCR7 peptides were used to determine dissociation constant (K_d_) values through nonlinear fitting to an equation that takes into account ligand depletion, as previously described [41,52].

## Figures and Tables

**Table 1:** CCR7 N-terminal modifications enhance CCL21 binding affinity.

CCR7	K_d_ (μM) ^1^ ± CI ^2^
5–11	700 ± 300
5–11 pY8	240 ± 60
5–11 sY8	140 ± 40
11–30	6,800 ± 500
11–30 pY17	1,700 ± 400
11–30 sY17	480 ± 70

^1^ K_d_ values here are from the fitting of the combination of all CCL21 residues that had significant chemical shift perturbations, as indicated in Figure 3. In the case of the CCR7 11–30 sY17, the K_d_ value is derived from only residues with a significant chemical shift that did not broaden beyond detection. ^2^ CI is the confidence interval at a 95% confidence level.

## Abbreviations

CCL21	CC chemokine ligand 21
CCL19	CC chemokine ligand 19
CCR7	CC chemokine receptor 7
NMR	Nuclear magnetic resonance
sY	Sulfotyrosine
pY	Phosphotyrosine
